# Comparison of software-assisted and freehand methods of rotational assessment for diaphyseal tibia fractures

**DOI:** 10.1007/s00590-025-04175-x

**Published:** 2025-01-27

**Authors:** Christian Blough, Kevin Huang, Samuel Raszka, Sapan Shah, John Garlich, Charles Moon, Geoffrey Marecek

**Affiliations:** 1 Cedars-Sinai Medical Centre, Los Angeles, USA; 2 University of Maryland Medical System, Baltimore, USA

**Keywords:** Tibia fracture, Rotational assessment, Software augmentation

## Abstract

**Objective:**

Accurate rotational reduction following tibial shaft fracture fixation is absent in up to 36% of cases yet may be critical for lower extremity biomechanics. The objective of this cadaveric study was to compare the results of freehand methods of reduction with software-assisted reduction.

**Methods:**

Four fellowship-trained orthopaedic trauma surgeons attempted rotational correction in a cadaveric model with fluoroscopic assistance (without radiographic visualization of the fracture site) using (1) their method of choice (MoC) and (2) software assistance (SA). After correction, deviation from baseline rotation was calculated.

**Results:**

The mean difference between the two methods (MoC–SA) was − 0.2° which was not statistically significant. There was no difference in variability between methods. The rate of clinically relevant rotational deformity (> 15°) was 28% using MoC and 31% using SA.

**Conclusion:**

Rotational assessment of diaphyseal tibia fractures in this cadaveric model was not significantly different when compared between method of choice and software augmentation.

## Introduction

The incidence of diaphyseal tibia fractures worldwide is high, estimated to be 16 per 100,000 persons per year [1]. The standard of care when managing diaphyseal tibia fractures in adults is intramedullary nailing. Although intramedullary nailing is generally a very successful procedure, inadequate reduction prior to, and during fixation, can lead to postoperative deformity associated with pain and decreased function.

Of the common deformities, rotation is the most difficult to assess clinically and radiographically [2]. A recent study revealed that the rate of noticeable postoperative malrotation (> 10 degrees of malrotation) may be as high as 36% [3]. If rotational alignment is not properly achieved, there may be risk for short-term complications like persistent pain and inability to perform desired activities, as well as long-term complications, like knee and ankle osteoarthritis [4–6]. These risks are unclear though, as the one study evaluating functional outcomes after tibial intramedullary nailing did not find a significant difference in mid-term outcomes with malrotation present [7]. There is a lack of knowledge regarding the long-term functional outcomes of patients with postoperative malrotation. None the less, rotational malalignment can have financial and medicolegal consequences for surgeons [8]. Despite our lack of data regarding functional outcomes in patients with postoperative malrotation, the goal should always be to restore native torsion.

While the importance of restoring rotational alignment is acknowledged, there is no clear consensus on the best method for achieving this. Many different intraoperative methods for assessing tibial rotation after intramedullary nailing are used today. One of the simplest is to compare the rotational alignment of the foot relative the contralateral limb. This method requires being able to visualize the contralateral extremity intraoperatively and assumes the other limb has not had a prior injury or torsional difference. Another method is the cortical step sign, in which the cortical thicknesses proximal and distal to the fracture are compared and matched. If performed correctly this method can be a reliable tool but has its limitations, as it is not reliable in comminuted fractures or with segmental bone loss [9]. In 1989 Clementz proposed an intraoperative method to assess tibial torsion, in which the rotation of a C-arm between a perfect lateral radiograph of the knee and a mortise radiograph of the ankle is calculated [10]. This method has found to be reliable but relies on intact tibiofemoral and tibiotalar relationships between extremities. Studies have shown that tibial torsional differences are prevalent between limbs in healthy adults [3]. Finally, in 2022 Roberts et al. described the intermalleolar method, in which the angle between a perfect lateral radiograph of the knee and radiograph of the ankle in which the fibula bisects the tibia at the level of the physeal scar is calculated and matched to the uninjured extremity [11]. This method has also been found to be reliable, but again relies on an uninjured contralateral extremity with similar tibial torsion [12]. Despite the recent advances in intraoperative assessment of tibial rotation, the rate of malrotation remains high, indicating the need for further investigation in this area.

A novel fluoroscopy-based software program (Surgeon Checklist Trauma, RadLink, El Segundo, California) can be used to assess rotation intraoperatively. The software can automatically detect bony landmarks and then overlay images with the bony landmarks outlined to assist in rotational reduction of fractures. If this software is able to improve surgeons’ ability to assess rotational alignment intraoperatively compared to current techniques, it could be a valuable tool in the management of diaphyseal tibia fractures.

The purpose of this study is to determine if software assistance can improve rotational reduction in diaphyseal tibia fractures compared to currently used techniques. We hypothesized that software assistance would improve rotational reduction because of the additional visual cues provided to the surgeon regarding the rotational alignment of the tibia.

## Materials and methods

### Specimen preparation

**Fig. 1:**
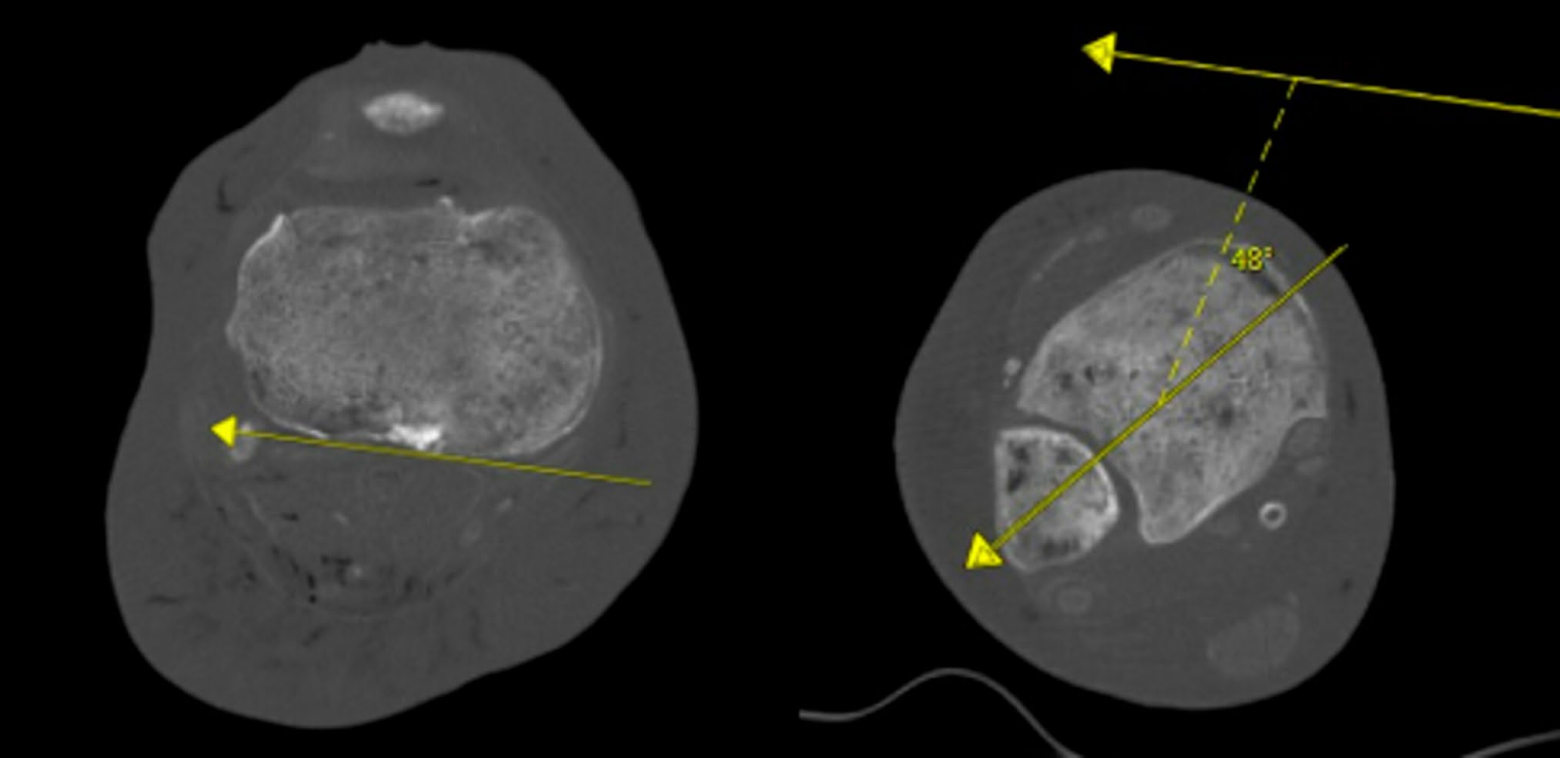
CT assessment of tibial torsion

Five matched pairs of knee to toe cadaveric specimens without history of lower extremity injury or surgery were obtained (Science Care, Phoenix, AZ). Each cadaveric lower extremity underwent computed tomography (CT) lower extremity rotational profile. The tibial torsion was then calculated using the method described by Jend et al. in 1981 (Fig. 1) [13].

**Fig. 2:**
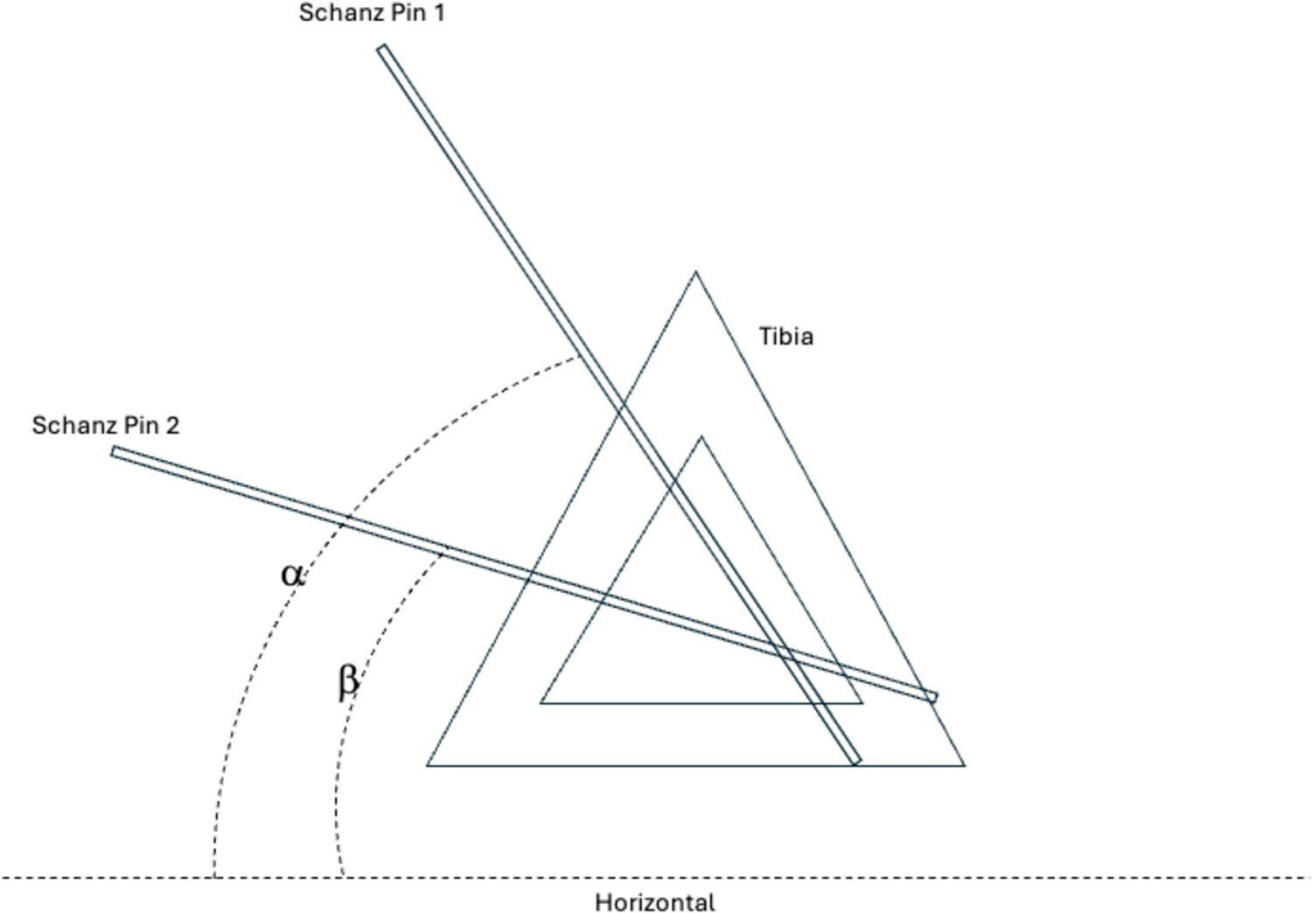
Baseline measurement between Schanz pins. Baseline = α–β

One side was randomly chosen to be the fractured extremity using a random number generator. Schanz pins were placed in the proximal and distal tibial metaphyses at random orientations in the axial plane. The angle difference from horizontal (i.e., the plane of the surgical table) of each Schanz pin was recorded with a magnetic electronic level (DL1, SanLiang, China) resting on each pin as seen in Fig. 2. The difference in angles was calculated and recorded as the baseline. Next, a transverse osteotomy was performed in the midshaft of the tibia to mimic a tibial shaft fracture. A fibular osteotomy was performed at the same level. A guidewire was then placed in an antegrade fashion into the medullary canal of the fractured tibia to simulate reduction with an intramedullary nail intraoperatively.

### Rotational correction

Four fellowship-educated orthopaedic traumatologists with a mean of 9 years experience (range 1–22 years) were prompted to adequately reduce the fracture in each specimen with two techniques. The first technique would be the method of their choice (MoC). The second technique would be using software assistance (SA). They were provided with a C-arm, an assistant for manipulating the C-arm, a tower containing a screen and the computer running the software program, as well as a representative to assist with the software. Surgeons were not permitted to visualize the fracture site fluoroscopically.

To begin each trial, the matched knee to toe cadaveric specimens were placed onto an operating room table. The rotation about the fracture on the fractured side was then randomly manipulated before the surgeon was allowed to enter the room, thus blinding them to the “correct” orientation of the Schanz pins. The surgeon was then asked to reduce the fracture using their MoC. All surgeons chose to use the intermalleolar method described by Roberts et al. as their MoC for assessing rotation [11]. This method has been validated in clinical practice [12]. When they were satisfied with their reduction the angle between each Schanz pin and horizontal was again measured and recorded. The rotational alignment was measured and compared to the baseline measurement prior to osteotomy. This was done for each of the five cadaver pairs. Between each trial the rotation of the Schanz pins was randomly manipulated with the treating surgeon out of the room.

Next, this protocol was repeated with each surgeon using SA to assist in their rotational reduction. Each surgeon chose anteroposterior (AP) fluoroscopic views of the proximal and distal tibia to assess rotation. A baseline shot of the intact proximal and distal tibia were taken and recorded. The cortices of bony landmarks about the knee and ankle from these fluoroscopic images of the intact limb were then outlined with the assistance of the software, which appear as colourful outlines on the image. This outline was mirrored to match the injured limb and displayed. The surgeon then attempted to match the AP view of the ankle on the injured limb with the displayed, outlined image (Fig. 2). Once satisfied with the fluoroscopic image of the injured limb, the image was uploaded to the software, and the cortices of bony landmarks surrounding the ankle on the injured limb were outlined and overlaid with the intact outline. If satisfied, the angle between each Schanz pin and horizontal was again measured in a similar fashion. If unsatisfied with the overlaid outline, they then corrected the rotational deformity of the injured side by matching the displayed, outlined view, of the intact side with the fluoroscopic view of the injured limb. This image was then again uploaded and the intact, outlined, side was overlaid. This process was completed until the surgeon was satisfied with the reduction. This was again done for each of the five cadaver pairs. Between each trial the rotation of the Schanz pins was randomly manipulated with the surgeon out of the room.

The rotational deformity of the injured tibia following rotational reduction was then compared to the ipsilateral, native ftibia prior to the simulates fracture, and the contralateral, matched tibia, using the difference in baseline torsion known from the baseline CT scanogram. Comparison to the contralateral tibia was done to simulate clinical practice, in which the uninjured contralateral tibia is available for comparison and is the surrogate for correction of the injured limb. We report both comparisons as we feel that it is important to evaluate rotational reduction methods relative to the native, pre-injured tibia, and the contralateral tibia which is often used as a surrogate for the native, pre-injure femur’s torsion.

### Statistical analysis

Surgeons performed each assessment twice. This totaled 40 reductions for the MoC and SA group each. The average measurement across the two study sessions was calculated for each specimen (tibia), surgeon, and method combination. This average rating was used for comparisons between methods, surgeons, and specimens. The rotation variables were identified as not normally distributed based on the Shapiro–Wilks test. For this reason, nonparametric tests were used to evaluate the difference in malrotation by method (Mann Whitney U test), by surgeon (Kruskal–Wallis test), and by specimen (Kruskal–Wallis test).

To assess clinically relevant differences categorical rotational deformity we performed Fisher’s Exact tests to compare across the two methods. Furthermore, we compared the variability of the two methods using an *F*-test.

## Results

The demographics and baseline characteristics of each tibia can be shown in Table 1. The average age of death for the donors was 75.2 ± 11.5 years old (range 55–89). Four (80%) of the donors were female and one was male. The average tibial torsion was 32.5° ± 10.5° (range 21–47°) of external rotation. The average difference (absolute value) in torsion between matched tibiae was 6.6° (range 1–20°.

**Table 1:** Demographics and baseline torsion

Specimen number	Sex	Age at death (years)	Left antetorsion (degrees)	Right antetorsion (degrees)	Antetorsion difference (Left–Right)
1 & **2**	Female	89	**45**	39	6
3 & **4**	Female	55	**37**	42	− 5
5 & **6**	Female	83	47	**27**	20
7 & **8**	Male	74	22	**23**	− 1
9 & **10**	Female	75	**22**	21	1

Bolded numbers are the “injured” laterality

When comparing relative the ipsilateral limb, evaluating restoration of native anatomy, the mean rotational deformity following reduction with MoC ranged from 8.8° to 15.8° by surgeon and from 8.1° to 16.5° by specimen. For SA the mean rotational deformity following reduction ranged from 7.2° to 18.3° by surgeon and from 7.4° to 15.3° by specimen (Table 2).

**Table 2:** Average rotational deformity

		Surgeon				Specimen				
		1	2	3	4	2	4	6	8	10
Vs. Ipsilateral	MoC	15.8°	11.5°	8.8°	10.0°	12.2°	16.5°	8.7°	8.1°	12.0°
	SA	18.3°	13.3°	7.2°	7.9°	15.1°	15.3°	8.6°	7.4°	12.1°
Vs. Contralateral	MoC	19.2°	15.3°	10.0°	8.5°	8.6°	17.2°	20.2°	8.1°	12.0°
	SA	21.7°	17.4°	10.9°	11.8°	13.2°	19.1°	26.2°	6.7°	11.6°

Average malrotation displayed as absolute value

When comparing relative to the contralateral limb, as is often done in clinical practice intraoperatively, the mean rotational deformity following reduction with MoC ranged from 8.5° to 19.2° by surgeon and from 8.1° to 20.2° by specimen. For SA the mean rotational deformity following reduction ranged from 10.9° to 21.7° by surgeon and from 6.7° to 26.2° by specimen (Table 2).

**Fig. 3:**
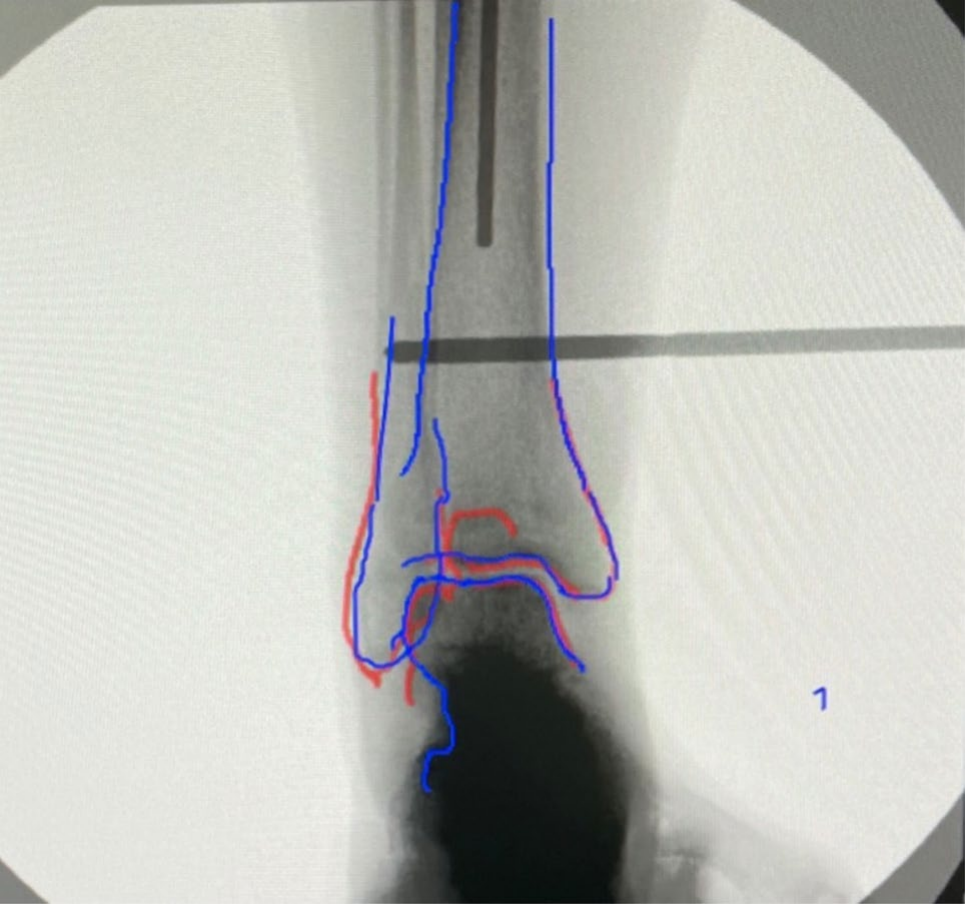
Software assistance cortical outline and overlay

**Fig. 4:**
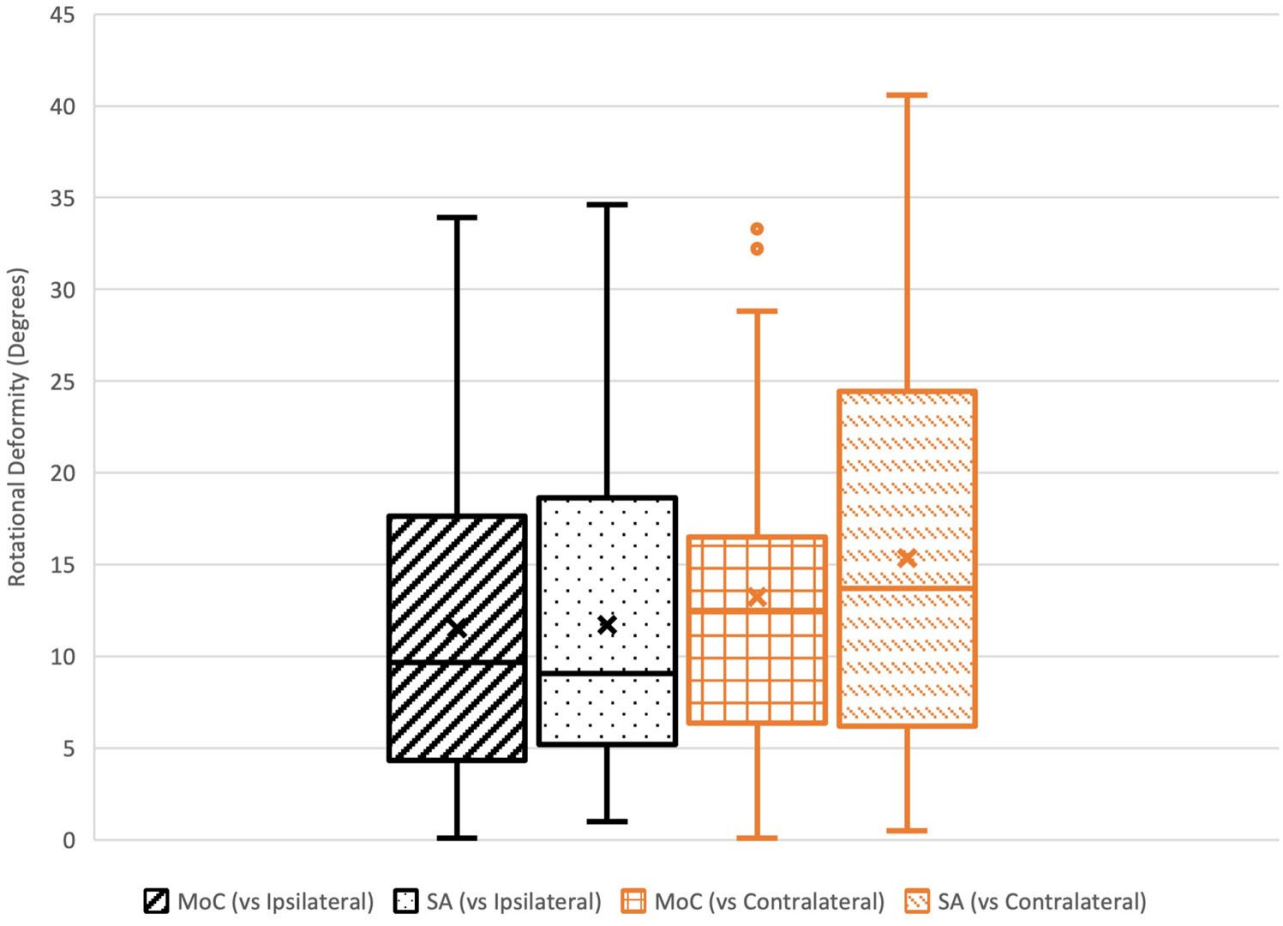
Rotational deformity by method (MoC—Method of Choice, SA—Software Assistance)

**Table 3:** Categorical comparison of rotational deformity

Malrotation (degrees)	Vs. ipsilateral		Vs. contralateral	
	Method of choice	Software-assistance	Method of choice	Software-assistance
	N (%)	N (%)	N (%)	N (%)
< 5	11 (28)	9 (23)	8 (20)	7 (18)
5.0–10	10 (25)	14 (15)	6 (15)	8 (20)
10.1–15	8 (20)	5 (13)	15 (38)	6 (15)
15.1–20	3 (8)	5 (13)	3 (8)	5 (13)
> 20	8 (20)	7 (18)	8 (20)	14 (35)

Absolute value of rotational deformity used for categorical comparison

The mean rotational deformity (absolute value) compared to the ipsilateral tibia after correction with MoC was 11.5° ± 8.4° (range 0.1° to 33.9°) and with SA was 11.7° ± 8.6° (range 1.0° to 34.6°); no significant difference was noted between methods (*p*-value 1). Relative to the contralateral femur, the mean rotational deformity (absolute value) after correction with MoC was 13.2° ± 8.7° (range 0.1° to 33.3°) and with SA was 15.4° ± 10.4° (range 0.5° to 40.6°); again, no significant difference was noted between methods (*p*-value 0.45). There was also no significant difference noted when comparing methods by surgeon or specimen (*p*-values > 0.05). The mean difference in rotational deformity (MoC–SA) relative to the ipsilateral femur was − 0.2° and relative to the contralateral femur was − 2.2°. Both of these differences were less than our calculated effect size. These results can be shown in Fig. 3.

Categorical comparison of rotational deformity by 5° intervals can be shown in Table 3. Fisher exact test results did reveal a significant difference between methods (MoC vs. SA) relative to ipsilateral or contralateral femur (*p*-values > 0.05).

In assessing the variability of rotational deformity between methods (MoC vs. SA), relative to the ipsilateral femur, the *F* test value was 0.94 (*p*-value 0.85) indicating no difference in variability of measurements between methods. Relative to the contralateral femur, the *F* test value was 0.69 (*p*-value 0.0.26), also indicating no difference in variability of measurements between method (Fig. 4).

## Discussion

Contrary to our hypothesis, software-assisted reduction produced comparable results relative to the intermalleolar for assessing torsional reduction of diaphyseal tibia fractures in this cadaveric study. These results should be reflective of the general population as our specimens had baseline tibial torsion within the established range of 27–35° [14, 15].

Described techniques to correct rotation about a diaphyseal tibia fracture include direct fracture reduction (visually), and indirect methods using fluoroscopy. The technique used is often dictated by fracture morphology, as a comminuted fracture is not amenable to direct fracture reduction or cortical alignment for rotational reductions. This study was designed to simulate a scenario in which rotational alignment cannot be obtained from direct measures, and instead necessitates indirect reduction. In 1989 Clementz described an indirect method to assess tibial torsion with intraoperative fluoroscopy, using the contralateral, uninjured limb as a surrogate [16]. More recently others have described other methods to assess tibial torsion with intraoperative fluoroscopy [11, 17]. All have been found to be reliable, yet the rate of rotational deformity remains high in practice.

Both the intermalleolar method and the SA method rely on an uninjured contralateral limb to assess rotation reduction. They also rely on the assumption that anatomic features and tibial torsion between limbs is consistent. Gallo et al. investigated the individual bilateral difference in tibial torsion with CT scans, finding an average of 5.3° in 195 patients. 12.3% of patients had > 10° individual bilateral difference. Caucasian patients were found to have a higher individual bilateral difference relative to Hispanic patients, but there was no correlation with age or sex [14]. Volkmar et al. similarly studies 229 patients with CT scans, finding an average individual bilateral difference in tibial torsion of 6.0°, with 18% of patients having a > 10° individual bilateral difference. They similarly found no correlation with age or sex [15]. Given that most rotational reduction techniques use the contralateral limb as a baseline to compare to, it is important to be aware of the possibility for significant individual bilateral difference in patients.

One of the specimen pairs (5–6) in this study had a 20° individual bilateral difference. When comparing to the contralateral limb for this specimen pair, both the intermalleolar and SA methods had higher mean rotational deformities relative to the other specimen pairs. This highlights the clinical relevance of individual bilateral difference in tibial torsion when assessing rotational deformity of diaphyseal tibia fractures.

Greater than 50% of reductions using each method led to a rotational deformity of greater than 10°, a rate higher than reported in previous literature [3, 7, 18].

RadLink’s “Surgeon Trauma Checklist” utilizes tracing of cortical bone landmarks on multiple fluoroscopic images, which can then be overlayed to assess for symmetry when obtaining fracture reduction. The software theoretically allows a more comprehensive rotational assessment given additional bone landmarks are traced besides just the ankle mortise or tibiotalar joint at the ankle. When using MoC 66% of reductions exceeded 10° of deformity, and while using SA 63% exceeded 10°, relative to the contralateral limb. Both of these rates are greater than the previously referenced rate in clinical practice of 36% exceeding 10° [3]. The reason for this may be due to the fact that in our study surgeons were unable to visualize the fracture site. This did not allow them to align the cortices about the fracture site or use the fibula as a surrogate for tibial rotation, both methods which are used in nearly every diaphyseal tibial fracture reduction.

The reason that software assistance did not produce superior rotational correction is unclear, but we have hypothesized multiple reasons. The first is that three of four traumatologists participating in this study had not previously used the software for assessing rotational correction of diaphyseal tibia fractures. There may be a learning curve that was not depicted in comparison of surgeons in this study. Another reason is that currently used methods may simply equivalent or superior.

There are several limitations to this study. The first is that it was completed on cadaveric specimens in which fractures were simulated. Second, the specimens underwent multiple freeze–thaw cycles, which may have affected the soft tissues. This could alter the ease of obtaining and maintaining reduction. Additionally, because of the degradation from multiple freeze–thaw cycles and the time-consuming nature of completing the study, we were limited to having four orthopaedic traumatologists complete the study. Although a study with a larger number of specimens and observers would be ideal, two recent studies used similar number of specimens with equal or fewer observers [11, 19]. Next, surgeons were provided a minimal introduction to the software package prior to use and some had not used the software during cases previously; a learning curve may be present that would allow improved results over time with software assistance. Finally, all surgeons in this study are traumatologists who routinely treat tibial shaft fractures. Other orthopaedic surgeons, who treat this injury less commonly, may produce different results in a similar study.

Another factor that is important when considering techniques to assess rotation reduction is the time needed to assess rotation and the amount of radiation that the patient and staff in the operating room are exposed to. Neither of these metrics were assessed in this study but should be included in future studies assessing SA.

Given the mean difference and standard deviation relative to the ipsilateral limb between methods found in our study (− 0.2°, ± 8.5°), we calculated that a study with 80% power to detect an alpha value of 0.05 would need over 28,000 reductions using each method to detect this effect size, using a sample size means calculation[20]. This indicates our study in underpowered, but a study of this size is likely not viable using a cadaveric model. We additionally calculated what number of reductions would be needed using each method to detect a clinically significant difference between methods of 15°. 7 reductions using each method was necessary to detect this difference; therefore our study with 20 measurements using each technique was adequately powered by this metric.

## Conclusion

Rotational reduction for diaphyseal tibia fractures treated with intramedullary nails can be challenging, with rotational deformity leading to short- and long-term complications. Software-assisted technology was investigated as a possible method to improve rotational assessment. In this cadaveric study, there was no difference found in the assessment of rotational reduction of diaphyseal tibia fractures with surgeons’ method of choice compared to software assistance. Given the high rate of tibial shaft fractures worldwide, further research in vivo is needed to determine the usefulness and applicability of software assistance for rotational assessment.

## Data Availability

De identified data is available on request.
